# Feasibility and predictors of change of narrative exposure therapy for displaced populations: a repeated measures design

**DOI:** 10.1186/s40814-020-00613-1

**Published:** 2020-05-21

**Authors:** Rina S. Ghafoerkhan, Henriette E. van Heemstra, Willem F. Scholte, Joriene R. J. van der Kolk, Jackie June F. ter Heide, Simone M. de la Rie, Linda M. Verhaak, Evelien Snippe, Paul A. Boelen

**Affiliations:** 1 ARQ National Psychotrauma Centre, Nienoord 5, 1112 XE Diemen, The Netherlands; 2 Department of Clinical Psychology, Utrecht University, Heidelberglaan 1, 3584 CS Utrecht, The Netherlands; 3 Dept. of Psychiatry, Amsterdam UMC, University of Amsterdam, Meibergdreef 9, 1105 AZ Amsterdam, The Netherlands; 4 Laguna Collective, Reigerstraat 16, 3816 AX Amersfoort, The Netherlands; 5 Sinai Centrum, Arthur van Schendelstraat 800, 3511 ML Utrecht, The Netherlands; 6 Department of Psychiatry, Interdisciplinary Center Psychopathology and Emotion Regulation, University of Groningen, University Medical Center Groningen, Hanzeplein 1, 9713 GZ Groningen, The Netherlands

**Keywords:** Refugees, Human trafficking, Sexual exploitation, Post-traumatic stress disorder, Narrative exposure therapy, Treatment response, Feasibility

## Abstract

**Background:**

Displaced victims of interpersonal violence, such as refugees, asylum seekers, and victims of sexual exploitation, are growing in numbers and are often suffering from a post-traumatic stress disorder (PTSD). At the same time, these victims are known to benefit less from trauma-focused therapy (TFT) and to be less compliant to treatment. The objective of this paper is to describe the rationale and research protocol of an ongoing trial that aims to evaluate different variables that might influence the feasibility of TFT for the study population. Specifically, perceived daily stress, emotion regulation, and mood are investigated as predictors of change in PTSD symptoms during a trauma-focused therapy (narrative exposure therapy (NET)). The feasibility of administering measures tapping these constructs repeatedly during treatment will also be evaluated.

**Methods/design:**

Using an observational treatment design, 80 displaced victims of interpersonal violence will be measured before, during, and after partaking in NET. Several questionnaires tapping PTSD plus the aforementioned possible predictors of PTSD change will be administered: Post-traumatic Stress Disorder Checklist-5, Perceived Stress Scale, Difficulties in Emotion Regulation Scale-18 (pre-test, post-test, and follow-up),subscale impulsivity of the Difficulties in Emotion Regulation Scale-18, Perceived Stress Scale short version, Primary Care Post-traumatic Stress Disorder and a single Mood item (each session). Multilevel modelling will be used to examine the relation between the possible predictors and treatment outcome.

**Discussion:**

The present study is the first to examine the interplay of facilitating and interfering factors possibly impacting treatment feasibility and effectiveness in displaced victims of interpersonal violence with PTSD receiving NET, using repeated measures. The current study can help to improve future treatment based on individual characteristics.

**Trial registration:**

Netherlands Trial Register: NTR7353, retrospectively registered. Date of registration: July 11, 2018.

## Background

Worldwide, there is an increase in the number of victims of interpersonal violence who are forced to leave their home country [1]. When entering a host country, these displaced persons are usually referred to as either ‘refugees’, ‘asylum seekers’, ‘trafficked human beings’, ‘illegal immigrants’ or ‘undocumented people’, depending on their trauma background and legal status. Mostly these groups overlap in their experience of forced migration, their marginalized social position, and the challenges they encounter. In this paper, we will therefore collectively refer to all such groups as ‘displaced people’.

As a result of assorted traumatic events, like war- and conflict-related violence, sexual violence and exploitation, many of these displaced people suffer from a post-traumatic stress disorder (PTSD) [2–5]. PTSD symptoms cause a great burden, render people unable to engage in daily activities and put them at risk for re-victimization [6]. Displaced people suffering from PTSD could therefore benefit from trauma-focused therapy (TFT). TFT is a psychological intervention aiming to decrease PTSD symptoms by fostering the processing of traumatic memories.

Scientific research into TFT for displaced people is scarce. Studies into TFT for non-refugee traumatized populations show higher effects (*d* = 1.08–1.40) [7], than for refugee populations (*g* = .25–1.01) [8]. Moreover, the feasibility of evidence-based therapies within the refugee population is complicated by post-migration obstacles [9] which can result in low treatment compliance and completion levels. Most likely, the primary focus on PTSD fails to address the broader challenges faced by displaced people [10]. Therefore, more insight is needed into factors affecting treatment response during TFT and conditions for recovery. Identifying factors that undermine the feasibility of TFT for displaced people may help to refine the timing and focus of interventions, and thus improve treatment response.

One key factor that potentially undermines the feasibility of treatment is daily stressors, given their proven association with PTSD symptoms [11–13]. Ongoing daily stressors arising from the immigration process, loss of social network, and impaired functioning resulting from PTSD symptoms negatively impact mental health among the study population [13–16]. The burden caused by ongoing daily stressors may impact one’s cognitive functioning in several ways [17]. For example, by occupying the working memory [18], and thereby reducing the cognitive resources needed to process traumatic events.

While there is evidence for the negative impact of daily stressors and perceived daily stress on treatment for displaced populations, the few present studies have yielded ambiguous results. One study affirmed the negative impact of lack of social support, a daily stressor, when present at the start of treatment on treatment outcome [19]. However, a study where clinician-rated daily stressors during treatment could not establish such an impact on treatment [20]. These limited and contradictive findings illustrate the need for more rigorous research on the relation between daily stressors and treatment response within the study population. No study to date has explored the impact of experienced daily stressors while taking part in TFT in the study population.

Another main factor likely to affect the feasibility of TFT for displaced people is emotion regulation, such as insight into, control over, and awareness of one’s emotions (e.g., [21, 22]). Several studies have indicated that emotion dysregulation mediates between traumatic events and the development of PTSD in different groups (e.g., [23]), including traumatized refugees [24]. Emotion dysregulation has been identified as a consequence of trauma and emotional impulsivity in particular as a predictor of future (re)victimization [25, 26]. For TFT to be feasibly applied, a person must be able to stay within a dynamic ‘window of tolerance’, a range of affect that can be regulated at that point in time [27]. Consequently, it is expected that the level of emotion regulation at baseline and improved control over one’s emotions during treatment are both prerequisites for reducing PTSD symptoms [28]. No study to date has looked into the interplay between emotion regulation and PTSD symptoms while taking part in TFT in the study population.

A final main factor that may affect treatment feasibility is depressed mood, which can be measured as a proxy for depression [29]. Among resettled refugees, the comorbidity rate for PTSD with depression is 44%; for depression with PTSD, it is 71% [2]. Previous research on TFT shows that higher baseline levels of depression predict poorer treatment response in displaced individuals [30] and therewith undermine the feasibility of treatment. Meanwhile, TFT has proven effectiveness in reducing symptoms of depression in the study population [31]. Yet, insight in the interaction between depressed mood and PTSD during treatment is currently lacking.

In an umbrella review of prevalence and intervention studies on common mental disorders in asylum seekers and refugees, Turrini et al. [32] found that narrative exposure therapy (NET) was the best-supported TFT for reducing PTSD symptoms. In a meta-analysis of NET [33], it was found that NET is effective in reducing PTSD and depression symptoms across diverse, predominantly war-affected refugee populations [34]. Since it is a first-choice treatment for the study population, NET was chosen as the TFT applied in the present study [33].

As outlined above, several factors (e.g. PTSD, perceived daily stress, emotion dysregulation and mood) tend to impact treatment feasibility. However, their interplay during TFT has not yet been examined. Insight in the feasibility of measuring this interplay in a diverse group of displaced individuals following NET is yet to be established. To best of our knowledge, there are no prior treatment studies for which weekly repeated measures have been carried out within the target population. Additionally, the practical execution of examining the interrelatedness between different parameters (e.g. PTSD, perceived daily stress, emotion dysregulation and mood) is based on questionnaires that are partly adapted for the current study (see ‘Methods’ section). Consequently, their feasibility within the target population has not been objectified yet.

In the present paper, the feasibility, rationale and protocol of an ongoing trial are described. Specifically, we aim to identify relevant predictors of PTSD symptom change during and after NET in 80 displaced victims of interpersonal violence. The primary hypotheses of the study are (1) it is feasible to administer highly repeated measures, within a diverse group of displaced persons. (2) High perceived daily stress, emotion dysregulation, and low mood at baseline and during NET predict higher drop-out, higher no-show and poorer treatment response of NET (i.e. less PTSD symptom reduction), thus undermining the feasibility of NET; (3) Reduction in perceived daily stress and improvement in emotion regulation and mood during NET are associated with concurrent reductions in PTSD symptoms during NET; (4) Change in perceived daily stress, emotion dysregulation, and mood during NET predict subsequent changes in PTSD symptoms at later stages of NET. Furthermore, the study aims to establish whether NET contributes to positive aspects of mental health.

## Methods

**Fig. 1:**
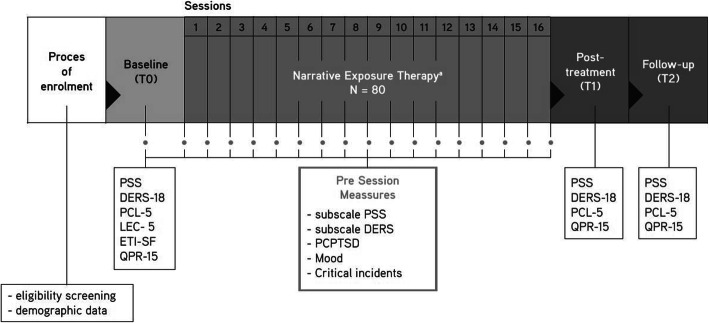
Design and overview of planned assessments. Note*:* Biographical data: gender, age, residence status, etc.; ETI, Early Trauma Inventory- Short Form; DERS-18, Difficulties in Emotion Regulation Scale-Short Version; LEC-5, Life Events Checklist for DSM-5; PCL-5, Post-traumatic Stress Disorder Checklist for DSM-5; PCPTSD, Primary Care Post-traumatic Stress Disorder; PSS, Perceived Stress Scale; QPR-15, Questionnaire on Process of Recovery-Short Version

This study follows a repeated measures observational design with baseline assessments, repeated measures over the course of treatment and post-treatment assessments. Twelve to sixteen sessions of NET will be provided to all patients included in the study. Various questionnaires will be administered at baseline (T0, pre-treatment), 1 week after NET completion (T1, post-treatment) and 6 weeks after NET completion (T2, follow-up). In addition, assessments to measure potential processes of change will be performed at the start of each NET session. See Fig. 1 for a detailed overview of the planned design and administered measures.

NET will be provided by trained psychologists, psychotherapists, medical doctors and psychiatrists. Psychologists and master’s level psychology students will perform assessments for the study. All involved professionals have extensive experience in working with migrants (in a culturally sensitive manner) and interpreters.

### Participants

The study will take place at an outpatient clinic specialized in mental health care for refugees, asylum seekers, victims of sexual exploitation and otherwise traumatized populations in the Capital region in the Netherlands. The study aims to include 80 participants, in order to detect medium to small effect sizes [35]. Patients are referred to the clinic by a general practitioner or by a partnering social welfare organization. Patients’ background/nationality is diverse; most originate from West Africa, the Middle East and Eastern Europe. Their legal status varies from having obtained a residence permit or even Dutch citizenship to illegal residence in the country, and they may reside in asylum seekers centres, specialized shelters for victims of sexual exploitation, governmental shelters for illegal persons or live independently.

#### Inclusion criteria

Patients will be included in the study if they are displaced victims of interpersonal violence, aged 18 years or older, if they have PTSD as a primary diagnosis established by a psychiatrist or clinical psychologist during intake, if individual NET in an outpatient setting has been indicated for them and if they are cognitively able to give informed consent to participate in the study.

#### Exclusion criteria

Patients will be excluded from the study if they display signs of an acute crisis, such as acute suicidality or acute severe psychosis, or suffer from persistent substance abuse. These exclusion criteria are applied because of their expected disturbing influence on the adherence and/or completion of NET.

#### Recruitment

This is an ongoing treatment trial. The enrolment period is planned to run for 2.5 years from February 2018 to January 2021. Following multi-disciplinary clinical assessment and evaluation, the diagnosis and treatment indication will be discussed with patients. Those who are eligible for enrolment will be informed about the study and will be invited to participate. This eligibility will be considered as broadly as possible within the inclusion criteria to allow for a representative sample of displaced victims of interpersonal violence. Information about the study aims and objectives, guarantee of anonymization of data and the fact that participants are free to terminate participation at any moment will be given orally and provided on paper through an information leaflet. After, intake patients are placed on a waiting list for NET, ranging from 1 to 4 months. When patients from the waiting list are invited to start NET, they will once again be informed about the study orally and on paper. Patients will be asked to consider participation over a minimum period of 3 days. Afterwards, they will be contacted by phone or face-to face and given ample opportunity to ask additional questions. If they indicate willingness to participate in the study, an appointment is made to sign informed consent and conduct the baseline assessment (T0). Participants are invited to bring someone they trust to this meeting. Participants will be receiving a voucher of 10 euros after the follow-up measurement. If after the first year the aspired total number of participants seems unachievable, prolongation of the enrolment period and multicentre options will be explored after consultation with the involved medical ethical committee.

### Intervention

#### Narrative exposure therapy

NET is an evidence-based short-term psychotherapy targeting PTSD symptoms, specifically appropriate for multiple traumas in divergent cultural settings. For the study population in question, the method is found to be feasible [36] and is considered the first choice TFT [33]. The NET protocol includes 12–16 sessions of individual trauma focused exposure, performed weekly or twice a week by trained mental health professionals. Each session lasts 90–120 min, depending on the content of the trauma which is targeted during the session, and the possible involvement of an interpreter. NET aims to create a chronological narrative of a patient’s life story, including both traumatic and empowering memories. During each session, one or more significant memories (traumatic or empowering) are discussed in great detail. Imaginary exposure, meaning-making, and reprocessing are used to reduce PTSD symptoms. An account of each session is written down by the therapist, which will result in a patient’s biography when the therapy has been completed.

#### Co-interventions before and during treatment

When participants cannot immediately start NET, there are no restrictions in psychological or pharmacological interventions received during their waiting period, applied in accordance with the national guidelines. Such interventions can be indicated either for symptom management or as a preparation of individual TFT. In addition, individual sessions can be provided in case of (imminent) crisis. From the start of NET onwards, however, no other modules will be provided, unless in case of acute crisis in which the safety of a participant is endangered or he/she is about to harm others. Previously started pharmacological interventions can be continued during NET; however, no new pharmacological interventions will be started during NET.

#### Discontinuation of the intervention and drop-out

Discontinuation of NET will take place if patients so wish or if multi-disciplinary clinical evaluation indicates that continuation is not in the best interest of the patient. If patients wish to stop participating in the study but wish to continue NET, this will be allowed. Patients will be considered dropouts when there are deviations from the NET treatment protocol for more than four consecutive sessions (i.e. no trauma-focused approach during the sessions or no-show), since in these cases the effectiveness of the treatment offered cannot be assured [34].

### Measurements

#### Perceived Stress Scale (PSS)—full scale administered pre-and post-treatment

The PSS [37] has been developed to measure the perception of daily stress by assessing how unpredictable, uncontrollable and overloading patients experience daily life. With 10 items, thoughts and feelings and the evaluation of daily life in the last month are explored through a 5-point scale ranging from 0 to 4. Mean scores will be calculated, ranging from 0 to 4. Example item: ‘In the last month, how often have you been upset because of something that happened unexpectedly?’ Administration of the questionnaire takes approximately 10 min. Acceptable psychometric properties of the instrument have been established [38].

#### Subscale administered before each session

The four items version of the PSS [39] will be administered at the start of each session. These items have proven sensitivity to short-term changes in stress [40]. For the purpose of this study, the indicated timespan was changed from ‘In the last month’ to ‘In the last week’ to match the other pre-sessions measures for possible predictors. An example item is ‘In the last week, how often have you felt that things were going your way?’ These items will be scored on a VAS-scale of exact 10 cm by placing a cross on a line from 0 (not at all) to 100 (completely). Mean scores will be calculated, ranging from 0 to 100.

#### Difficulties in Emotion Regulation Scale short version–18 (DERS)—full scale administered pre- and post-treatment

The DERS was developed to measure emotion regulation. The DERS-18 comprises 18 items and uses a 5-point Likert scale ranging from 1 to 5; it has 6 subscales: non-acceptance of emotional responses, difficulty engaging in goal-directed behaviour, impulse control difficulties, lack of emotional awareness, limited access to emotion regulation strategies and a lack of emotional clarity. Mean scores will be calculated, ranging from 1 to 5. Example item: ‘I pay attention to how I feel’*.* Administration of the questionnaire takes approximately 10 min. The DERS has high internal consistency, good test-retest reliability, moderate construct and predictive validity [41].

#### Subscale ‘impulsivity’ administered before each session

As a proxy of emotion regulation, the subscale ‘Impulsivity’ will be used, comprising three items from the ‘Difficulties in Emotion Regulation Scale’ (DERS-18; 40). For the purpose of this study, the phrase ‘In the last week I have felt:’ was added to match the other pre-session measures for possible predictors. Example item: ‘When I am upset, I become out of control’. Items will be scored on a VAS-scale ranging from 0 (not at all) to 100 (completely)*.* Mean scores will be calculated, ranging from 0 to 100.

#### PTSD Checklist for DSM-5 (PCL-5)—administered pre-and post-treatment

The 20 item PCL-5 is a self-report checklist which measures the presence and severity of the 20 DSM-5 symptoms of PTSD on a 5-point scale (0–4) (e.g. ‘Trouble remembering important parts of the stressful experience’)*.* Mean scores will be calculated, ranging from 0 to 4. It will be used to indicate PTSD symptom severity pre- and post-treatment. Administration of the questionnaire takes approximately 10 min. The instrument has good psychometric quality [42].

#### Primary Care Post-traumatic Stress Disorder (PCPTSD)—administered before each session

Because a short version of the PCL-5 is unavailable, the five-item Primary Care Post-traumatic Stress Disorder (PCPTSD) checklist was selected to increase the feasibility of the frequently repeated measurements. This questionnaire is used to measure PTSD symptoms [43]. For the purpose of this study the indicated timespan was changed from ‘In the past month, have you’ to ‘In the last week’ to match the other pre-session measures for possible predictors. An example item is: ‘In the last week did you have nightmares about the event(s) or thoughts about the event(s) when you did not want to?’ These items will be scored on a VAS-scale ranging from 0 (not at all) to 100 (extremely). The questionnaire has good psychometric qualities [41]. Mean scores will be calculated, ranging from 0 to 100.

#### Mood—measured before each session

Mood will be measured using a validated single item measure [44]. For the purpose of this study, we have altered the item ‘At the moment I feel’ to ‘In the last week I felt’ to make the timespan congruent to the other pre-session measures for possible predictors. This mood item will be scored on a VAS-scale ranging from 0 (sad) to 100 (happy). Mean scores will be calculated, ranging from 0 to 100.

#### Critical incidents

Before each therapy session, the client will be asked to report if any relevant personal circumstances have arisen since the last appointment.

#### Other

Biographical data of patients will be collected (i.e. gender, age, educational level, current residence, legal status, country of birth) to describe the study population.

#### Questionnaire on Process of Recovery Short Version–15

The QPR is a 5-point scale (0-4), self-report questionnaire that probes people’s recovery and meaningful aspects in the recovery process (e.g., ‘I feel able to take chances in life.’). The QPR-15 consists of 15 items. Mean scores will be calculated, ranging from 0 to 4. The questionnaire has good psychometric properties, and has proven to be associated with quality of life, empowerment, and psychological wellbeing [45]. Administration of the questionnaire lasts approximately 20 minutes. The questionnaire has been selected because it represents psychological wellbeing beyond the scope of mental health symptoms. The QPR is reliable and valid, and has proven to be associated with quality of life, empowerment and psychological wellbeing [45].

#### Early Trauma Inventory–short version

The Early Trauma Inventory–short version (ETI-SF) was developed to determine potentially traumatic events before and after the age of 18 years old [46]. The ETI-SF comprises 27 items that assess physical, emotional, and sexual abuse. Items vary between open questions and multiple choice being answered with yes or no. The scale has a range from 0 to 29, with higher scores referring to a higher number of traumatic experiences. Administration of the questionnaire lasts approximately 10 min. Its reliability and validity are good [47]. The questionnaire has been selected to describe trauma-related features of the study population.

#### Life Events Checklist (LEC–5)

The LEC-5 comprises 17 multiple choice items on a 6-point nominal scale (i.e., ‘happened to me’, ‘witnessed it’, ‘learned about it’, ‘part of my job’, ‘not sure’, ‘doesn’t apply’). The checklist aims to determine whether someone has ever been exposed to 16 events known to potentially result in PTSD or distress, and one additional event not captured in the first 16 items. In the last part of the LEC-5, respondents are asked which event he/she considers to have had the most impact, followed by 7 questions, both open- and multiple-choice questions, aimed at identifying the characteristics of this event. The scale has a range from 0 to 68, with higher scores referring to a higher number of traumatic experiences. Administration of the questionnaire lasts approximately 20 min. The reliability of the LEC-5 is considered to be good [48]. The questionnaire was selected to describe trauma-related features of the study population.

### Data collection, management and analysis

#### Procedure pre-session measures

During baseline (T0) and at the start of each TFT session, the patient’s fixed practitioner or a supervised master’s level psychology student will administer the thirteen items selected to assess the four abovementioned possible predictors. To avoid an order effect, the sequence of the measures for possible predictors and the order of questions within every measure will change with every session. These measures will only be administered during the treatment period. They will be administered by pen and paper and are expected to take on average 10 min. Questionnaires are translated from their original English version according a forward-backward procedure by two independent native speakers into Dutch, and by professional translators into Arabic, French, Amharic and Tigrinya. Interpreters will assist for other languages.

#### Procedure pre- and post-treatment

During baseline (T0), post-treatment (T1) and follow-up (T2) measurements, the abovementioned set of questionnaires will be administered. See Fig. 1 for a detailed overview of the planned time points for assessments. These questionnaires will be administered digitally and are expected to take a maximum of 80 min. Questionnaires are available in Dutch and English. Given the lack of computer skills and the often limited Dutch and/or English proficiency within the study population, a clinician and interpreter will be available for assistance. Measurements will take place at the treatment location; if preferred, this meeting will be combined with other appointments. After the last assessment, participants will receive a voucher of 10 euros.

### Statistical analysis

First, multiple regression analyses and logistic regression analyses will be performed to examine whether high perceived daily stress, emotion dysregulation and low mood at baseline predict drop-out, no-show and treatment outcomes. Secondly, the concurrent association between each possible predictor measured prior to each session (i.e. perceived daily stress, emotion regulation and mood) and PTSD symptoms will be examined using bivariate (multilevel) growth modelling, as multiple observations of the predictor variables and PTSD symptoms (level 1) are nested within individuals (level 2) [49]. In order to infer that change in the predictor variables leads to change in PTSD symptoms, we will examine the timeline (i.e. temporal precedence) in two ways [50, 51]. The dynamic (i.e. temporal) associations will be examined by estimating whether change in PTSD symptoms from the previous week to the current week (t) can be predicted by change of a possible predictor at the previous week (t-1) using multilevel modelling. To examine the timeline of larger shifts instead of week-to-week changes, it will be examined whether the earliest significant decrease in mean levels of each possible predictor (improvement) occurs before the largest reduction in mean levels of PTSD symptoms.

## Discussion

Among the growing number of forcibly displaced people worldwide, many are suffering from trauma-related mental health problems. As psychological treatments for displaced persons with PTSD appear to be less successful than for other populations, insight is needed in factors that affect the feasibility of these treatments. However, relevant research is lacking. The ongoing trial presented in this paper is the first to examine the interplay of factors for feasibility and effectiveness of in displaced victims of interpersonal violence receiving NET. More specifically, the impact of perceived daily stress, emotion dysregulation, and disturbed mood on PTSD symptom changes during TFT are examined.

The theoretical basis of NET has been well documented, and its effectiveness has been examined in various studies and contexts [33, 34, 36]. Yet, specific factors contributing to positive outcomes or constraining its feasibility are largely unidentified. In the current study, repeated measures will identify various constructs relevant for treatment feasibility and response of NET. Since, to our knowledge, this is the first study to use repeated measures within the target population, findings will additionally provide insight into the feasibility of this method for displaced populations.

As the method requires only minor adaptations to usual treatment proceedings and follows an observational design, participants can be included that might have to be excluded in more complex designs [52]. This allows for conclusions based on a sample representing the intended population with high external validity, which is exceptional for this specific group [53]. Although our design favours generalizability of the results, it may reduce the internal validity of the study; for instance, the present design does not allow for the examination of the extent to which observed changes in PTSD can be attributed to NET, but to factors such as natural recovery, bias or confounders. However, as we aim to study factors promoting or constraining treatment feasibility, a naturalistic design is the most ethical, feasible and time-efficient design to obtain answers to our research questions. Besides, the reduced internal validity is partially addressed by limiting the possibly confounding role of concurrent treatment.

Evidently, there are factors possibly impacting treatment response. For the purpose of this study, repeated measures were selected on the basis of relevant studies [41, 44] and clinical insights. Thus, the set of questions have been tailored to the specific characteristics of the study population. Conclusions drawn from the study therefore may hold relevant implications in clinical practice for displaced victims of interpersonal violence. Although the chosen subscales support the clinical validity of the study, the PTSD and emotion dysregulation subscales have not been validated as weekly repeated measures.

It is important to note that inclusion for the current study has already started; however, it is planned to run until early 2021. Critically, no data analysis has currently been performed yet.

We expect that the findings of this study will contribute to both the scientific and the clinical field. Identifying factors that limit treatment response may in turn inform the development of improved treatment modules that specifically address these blockages. The promotion of self-efficacy or problem-solving skills prior to NET, for example, may decrease perceived daily stress and its constraining effect on the feasibility of NET. Likewise, promoting emotion regulation prior to NET may intensify its potential supportive role for TFT. Additionally, if these factors have no predictive value for the course of PTSD during treatment, clinicians might be encouraged to offer TFT to patients despite the presence of daily stressors, emotion regulation difficulties and mood problems. By identifying predictors of treatment response, the current study can enable treatment indications to be tailored to individual characteristics.

Furthermore, exploring these predictors sheds a broader light on the consequences of trauma, beyond a narrow focus on PTSD, and may provide clues for a wider range of relevant treatment foci. In this way, this study responds to the ongoing debate on the emphasis focus of PTSD in designing treatments for displaced populations [10].

## Data Availability

The datasets generated and/or analyzed during the current study are not publicly available due to confidentiality of the patient data but are available from the corresponding author on reasonable request. Scientific papers will be written based on the collected data. For secondary data-analysis, the first authors can be contacted.

## Abbreviations

ETI: Early Trauma Inventory DERS Difficulties in Emotion Regulation Scale
LEC: Life Events Checklist NET Narrative exposure therapy
PCL: PTSD Checklist for DSM-5
PCPTSD: Primary Care Post-traumatic Stress Disorder
PSS: Perceived Stress Scale
PTSD: Post-traumatic stress disorder
QPR: Questionnaire on Process of Recovery
TFT: Trauma-Focused Therapy
