# Correction to: Feasibility and predictors of change of narrative exposure therapy for displaced populations: a repeated measures design

**DOI:** 10.1186/s40814-020-00651-9

**Published:** 2020-08-03

**Authors:** Rina S. Ghafoerkhan, Henriette E. van Heemstra, Willem F. Scholte, Joriene R. J. van der Kolk, Jackie June F. ter Heide, Simone M. de la Rie, Linda M. Verhaak, Evelien Snippe, Paul A. Boelen

**Affiliations:** 1 ARQ National Psychotrauma Centre, Nienoord 5, 1112 XE Diemen, The Netherlands; 2 Department of Clinical Psychology, Utrecht University, Heidelberglaan 1, 3584 CS Utrecht, The Netherlands; 3 Department of Psychiatry, Amsterdam UMC, University of Amsterdam, Meibergdreef 9, 1105 AZ Amsterdam, The Netherlands; 4 Laguna Collective, Reigerstraat 16, 3816 AX Amersfoort, The Netherlands; 5 Sinai Centrum, Arthur van Schendelstraat 800, 3511 ML Utrecht, The Netherlands; 6 Department of Psychiatry, Interdisciplinary Center Psychopathology and Emotion Regulation, University of Groningen, University Medical Center Groningen, Hanzeplein 1, 9713 GZ Groningen, The Netherlands

**Correction to: Pilot and Feasibility Studies 6, 69 (2020)**

**https://doi.org/10.1186/s40814-020-00613-1**

Following publication of the original article [1], the authors reported an error in the following sentence:

*Studies into TFT for non-refugee traumatized populations show higher effects (d = 1.08—1.40) [7], than for refugee populations (g = .25—1.01) [8].*

Which will be replaced by:

*There is evidence that non-refugee traumatized populations benefit more from TFT than traumatized groups with a refugee background [7, 8].*

This also involves changes in reference [8].

“Turrini, G., Purgato, M., Acarturk, C., Anttila, M., Au, T., Ballette, F., ... & Hall, J. (2019). Efficacy and acceptability of psychosocial interventions in asylum seekers and refugees: systematic review and meta-analysis. *Epidemiology and psychiatric sciences*, *28*(4), 376–388.doi.org/10.1017/S2045796019000027”.

should replace:

“Lambert JE, Alhassoon OM. Trauma-focused therapy for refugees: Meta-analytic findings. Journal of counseling psychology. 2015;62(1):28. DOI: 10.1037/cou0000048”.

The publishers apologise for this error.
